# Prediction of unilateral prostate cancer by the combination of transrectal ultrasonography-guided prostate biopsy and multi-parametric magnetic resonance imaging: A real-life experience

**DOI:** 10.1371/journal.pone.0202872

**Published:** 2018-08-29

**Authors:** Jung Jun Kim, Taejin Kim, Hakjong Lee, Seok-Soo Byun, Sang Eun Lee, Gheeyoung Choe, Sung Kyu Hong

**Affiliations:** 1 Department of Urology, Seoul National University Bundang Hospital, Seongnam, Korea; 2 Department of Radiology, Seoul National University Bundang Hospital, Seongnam, Korea; 3 Department of Urology, Seoul National University College of Medicine, Seoul, Korea; 4 Department of Pathology, Seoul National University Bundang Hospital, Seongnam, Korea; Public Library of Science, UNITED KINGDOM

## Abstract

Hemi-ablation of prostate cancer (PCa) requires an accurate prediction of laterality. Recently, multi-parametric magnetic resonance imaging (mpMRI) has recently been increasingly used to enhance clinical staging and characterization of tumor foci. Thus, we tried to investigate the real-life accuracy of combinatory approach of using both transrectal ultrasound (TRUS)-guided prostatic biopsy and mpMRI in predicting the laterality of PCa. We reviewed the records of 335 men who were suspected of having unilateral PCa on multi (≥ 12)-core TRUS-guided biopsy and preoperative mpMRI and subsequently had undergone radical prostatectomy (RP) at our institution. Based on the analysis of pathologic outcomes from RP, the performance of the combinatory approach in predicting the pathological laterality of PCa was evaluated. Pathology was classified to be unfavorable when showing a Gleason pattern of 4/5 or pT3/N1 features. Significant cancer was defined as non-organ-confined disease, having a Gleason pattern of 4/5, or showing a cancer volume of ≥ 0.5 mL. Among the 335 unilateral lobes not suspected to harbor tumor from either the TRUS biopsy or mpMRI, the actual absence rate of malignancy was only 13.7% from a pathologic analysis of RP specimens. Even among the 115 D’Amico low-risk group, the absence rate of malignancy was only 26.1% in unilateral lobes not suspected to harbor tumor. Among the 335 lobes, unfavorable pathology and significant cancer were not observed in 36.1% and 30.7%, respectively. The absence rates of unfavorable pathology and significant cancer among the D’Amico low risk group were 56.5% and 47.8%, respectively. Meanwhile, the absence rate of dominant Gleason pattern 4 or 5 was 74.9% among the 335 total subjects. Our real-life clinical experience showed that the combination of multi-core TRUS-guided biopsy and mpMRI did not provide reliable accuracy in the prediction of true unilaterality of PCa.

## Introduction

A widespread implementation of prostate-specific antigen (PSA) screening has contributed to earlier detection of prostate cancer (PCa) at lower stages, lower grades, and smaller tumor volumes [1]. Moreover, with advancements in minimally invasive treatment modalities, a wide variety of organ-sparing focal therapies for PCa have been introduced with the goal of minimizing complications [2]. Although focal therapies, such as high-intensity focused ultrasound (HIFU) and cryoablation, have not yet been regarded as the standard treatment modalities that can replace radical prostatectomy (RP) or radiation therapy, they have been acknowledged as having great potential. During the past decade, several reports have shown promising data regarding these organ-sparing therapies for localized PCa [3–6].

Meanwhile, a proper selection of candidates for focal therapy remains a major challenge in actual clinical practice. The success of focal therapy heavily depends on an accurate localization of significant tumor foci within the prostate. For hemi-ablation of PCa, accurate prediction of laterality of PCa is crucial. Traditionally, transrectal ultrasound (TRUS)-guided prostatic biopsy has been widely used to assess the laterality of tumor foci; however, the method was less than satisfactory with respect to its accuracy. To overcome such a limitation, multi-parametric magnetic resonance imaging (mpMRI) has been increasingly used to enhance clinical staging and characterization of tumor foci [7]. Recently, MRI-targeted biopsy has been advocated in hopes of preventing unnecessary biopsy and/or decrease the number of cores obtained to minimize patient burden [8]. However, the actual degree of accuracy of mpMRI remains controversial [9]. Thus, we tried to investigate the real-life accuracy of clinical staging using TRUS-guided prostatic biopsy and mpMRI, which is currently widely used in clinical setting, in predicting the laterality of PCa.

## Materials and methods

### Ethics statement

This retrospective study was approved by the Institutional Review Board (IRB) of Seoul National University Bundang Hospital (Seongnam, Republic of Korea). The approval number is B1706/402-115. The requirement for informed consent was waived. Personal identifiers were all removed and the data were anonymously analyzed.

### Patients

Between January 2010 and July 2015, a total of 730 patients were diagnosed with localized PCa (clinical T1/T2) via extended multi (≥ 12)-core TRUS-guided biopsy, underwent preoperative mpMRI with ADC mapping, and subsequently received radical prostatectomy (RP) at a single tertiary center. Of these 730 patients, 335 men-who were suspected of having unilateral localized prostate cancer from preoperative clinical staging onmulti (≥ 12)-core TRUS-guided biopsy and mpMRI, separately, and eventually underwent RP without any prior treatment were finally included in the present study. The pathological analyses including histopathologic mapping by quadri-mount section were performed in all 335 patients following RP. Predictive performance of the combination of pretreatment clinical parameters (multi (≥ 12)-core TRUS-guided biopsy, and mpMRI) in predicting the pathological laterality of PCa was evaluated.

### Imaging protocol

MRIs were performed using a 3.0-T MR system (Intera Achieva 3.0T, Philips Medical Systems, Best, The Netherlands) equipped with phased-array cardiac 6-channel coil. Thirty minutes prior to imaging, all patients were injected with 20 mg butylscopolamine (Buscopan, Boehringer Ingelheim Pharma, Ingelheim, Germany) intramuscularly to suppress bowel peristalsis. Axial diffusion-weighted (DW) images were acquired using single-shot echo planar imaging. The scan parameters were as follows: TR, 2,500–3,000 ms; TE, 56–65 ms; slice thickness, 3 mm; interslice gap, 1 mm; field of view, 180 mm×180 mm; matrix, 92×90; and number of excitations, 10. Diffusion encoding gradients were applied as a bipolar pair at b-values 0 and 1,000 s/mm2. ADC maps were automatically generated on a pixel-by-pixel basis. MRI examinations were conducted at least 2 weeks after prostate biopsy.

### Image analysis

Two experienced uro-radiologists with more than 10 years of mpMRI experience, who were blinded to all clinical variables including pathological outcome, graded the level of suspicion for PCa from mpMRI using the Prostate Imaging Reporting and Data System Version 2 (PI-RADS V2) scale from 1 to 5 in following manner: grade 1, highly unlikely to be present; grade 2, unlikely to be present; grade 3, equivocal; grade 4, likely to be present; and grade 5, highly likely to be present) [10]. MpMRI was considered negative in a unilateral prostatic lobe when no suspicious lesion was seen or when PIRADS grade was ≤ 2 in that lobe.

### Histopathology acquisition and analysis

For pathological examination, the surgical specimens were routinely fixed in formalin and embedded in paraffin following the standard protocols at our hospital. The paraffin-embedded tissue samples were cut in 4–6-μm-thick sections on a rotary microtome and subsequently stained with hematoxylin and eosin. Each slice was marked according to tissue orientation, annotated by slice number, fixed, and then paraffin embedded. Subsequently, 5-micron mount sections were cut from the paraffin-embedded tissue, mounted on glass slides, and stained with hematoxylin and eosin. The interface of the peripheral zone and transition zone, as well as all cancer foci were outlined with a marker on the glass slides by a genitourinary pathologist. Each cancer focus was assigned a Gleason score, calculated as the most predominant Gleason pattern plus the second most predominant Gleason pattern. A third Gleason pattern representing a minor tertiary component was included as the third grade, if and when present. In addition, the estimated percent of Gleason patterns 3, 4, and 5 were documented.

Two urological pathologists reviewed the stained sections and reported the final pathological results. The pathological stages and Gleason grades were determined according to the seventh edition of the American Joint Committee on Cancer staging system and the International Society of Urological Pathology, respectively [11] [12]. The whole mount section pathology, including histopathological topographic mapping reports after RP, was used to obtain information about the pTNM stage and Gleason score as well as tumor size and location.

Pathology was classified to be unfavorable when showing a Gleason pattern 4/5, or pT3/N1 features. [13]. Significant cancer was defined as non-organ-confined disease, having Gleason pattern 4/5, or showing a cancer volume of ≥ 0.5 mL [14].

### Statistical analysis

We first assessed the clinical characteristics of the cohort and the pathological outcome of the unilateral lobe not suspected to harbor tumor. The pathological outcomes were compared between the clinically low-risk group and high-risk group. Sensitivity analysis was performed to evaluate the accuracy of the combination approach in predicting the pathological outcome of each prostate lobe. Chi-square test was used for the comparison of pathological outcome and predictive performance. All statistical analyses were performed using SPSS 22.0 for Windows (IBM^®^ SPSS^®^ version 22.0, IBM, Armonk, New York, USA). The p values were considered statistically significant when <0.01.

## Results

**Fig 1:**
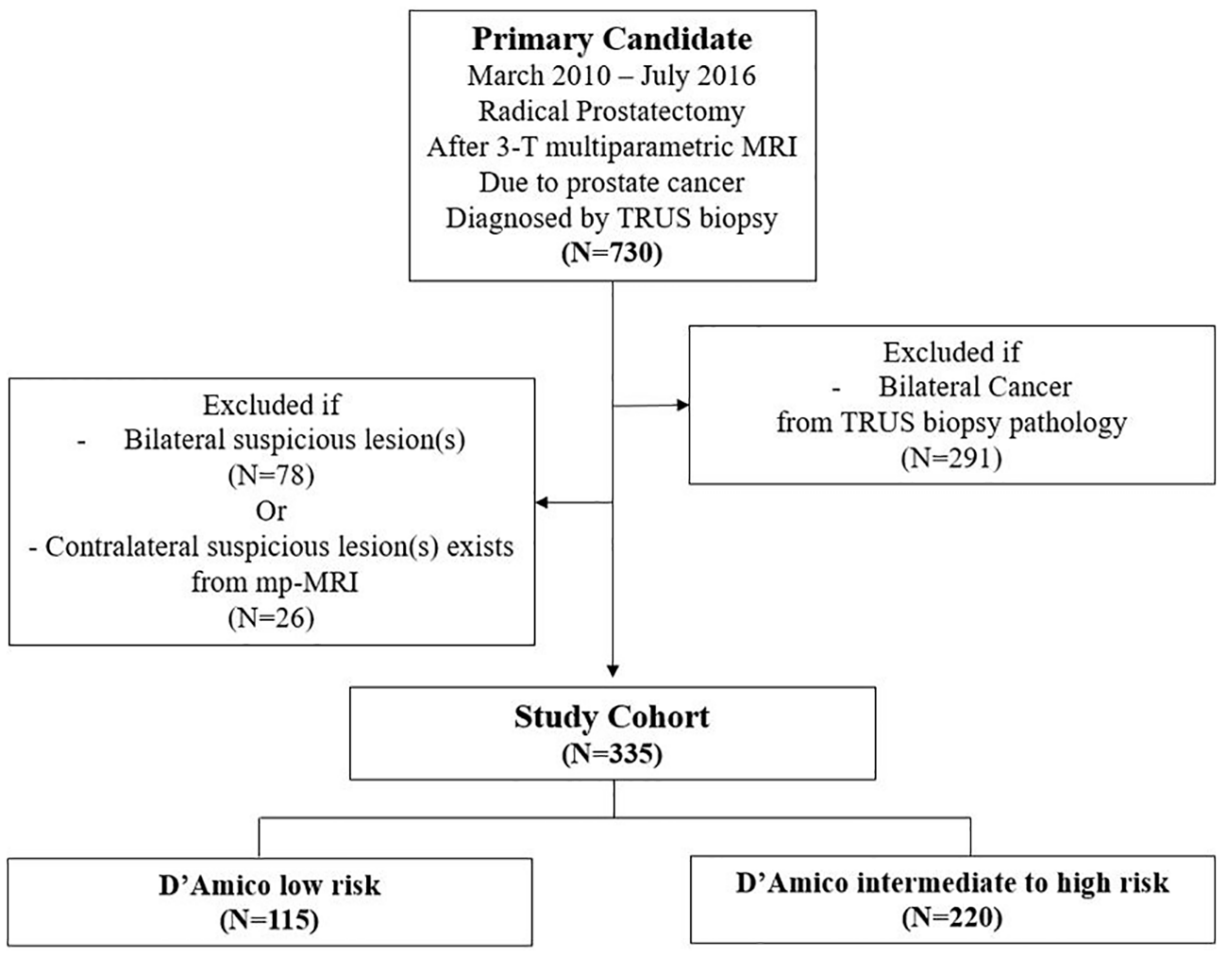
Patient selection flow diagram. MRI; Magnetic Resonance Imaging, DRE; Digital Rectal Exam.

Of the 730 patients who underwent TRUS-guided biopsy, mpMRI, and subsequently RP for the treatment of localized prostate cancer (T1/T2) at our institution between January 2010 and July 2015, there were 355 (45.9%) potential candidates for hemi-ablation who were suspected of having tumor only in the unilateral prostatic lobe from preoperative clinical staging (TRUS-guided biopsy, and mpMRI), as shown in Fig 1. The clinicopathological characteristics of these 335 men are summarized in Table 1. The median age and PSA were 65.1 years (45–82) and 7.8 ng/dl (2.2–21), respectively. Regarding the pathological outcome, among the 335 men, 34.3% had D’Amico low risk disease. The rates of locally advanced disease and local lymph node metastasis were 8.9% and 1.3%, respectively.

**Table 1:** Clinicopathological characteristics of patients with unilateral prostatic lobe not suspected to harbor PCa by the combination of TRUS biopsy and mpMRI.

Characteristics	Value	%
Number of patients	335	
Age (yr, range)	65.1 (45–82)	
PSA (ng/ml, range)	7.8 (2.2–21)	
Prostate volume (cc)	38.4 (12–64)	
Clinical stage		
T1b	1	0.3%
T1c	269	80.3%
≥T2	66	19.7%
Biopsy Gleason score		
≤6	193	57.6%
3 + 4	79	23.6%
4 + 3	40	11.9%
≥8	23	6.9%
Number of positive core		
≤2	143	42.7%
3–4	155	46.3%
≥4	37	11.0%
D’Amico risk group		
Low	115	34.3%
Intermediate	185	55.2%
High	35	10.4%
Pathological Stage		
T2a	26	7.8%
T2b	20	6.0%
T2c	259	77.3%
T3a	21	6.3%
T3b	9	2.7%
Lymph node status		
Nx	56	16.7%
N0	274	81.8%
N1	5	1.5%
Pathological Gleason Score		
3 + 3	52	15.5%
3 + 4	185	55.2%
4 + 3	88	26.3%
4 + 4	9	2.7%
Gleason Score up/downgrade		
Upgrade	172	51.3%
Downgrade	7	2.1%

Pca; prostate cancer, TRUS; transrectal ultrasonography, mpMRI; multiparameteric magnetic resonance imaging

Table 2 shows the pathological outcomes of unilateral prostate lobes not suspected to harbor tumor from TRUS-guided biopsy and preoperative mpMRI. Among the 335 unilateral lobes, the absence rate of malignancy was only 13.7%. In 3.0% of patients, the extraprostatic extension and/or seminal vesicle invasion were observed on the unilateral side with no preoperative tumor suspicion.

**Table 2:** Pathological outcome of unilateral lobe not suspected to harbor PCa by the combination of TRUS biopsy and mpMRI.

Characteristics	Value	%
Number of non-suspicious lobes	335	
No malignancy	46	13.7%
Pathological Stage		
T2	279	83.3%
T3a	6	1.8%
T3b	4	1.2%
Pathological Gleason score		
3 + 3	75	22.4%
3 + 4	130	38.8%
4 + 3	80	23.9%
4 + 4	4	1.2%
Tumor location		
Transitional Zone	131	39.1%
Peripheral Zone	212	63.3%
Tumor volume (mL)		
Global tumor volume		
Median (range)	0.86 (0.03–9.8)	
<0.5, n (%)	93	27.8%
≥0.5, n (%)	196	58.5%
Main tumor volume		
Median (range)	0.61 (0.03–20.7)	
<0.5, n (%)	132	39.4%
≥0.5, n (%)	157	46.9%
Number of tumors		
1	97	29.0%
≥2	192	57.3%

Pca; prostate cancer, TRUS; transrectal ultrasonography, mpMRI; multiparameteric magnetic resonance imaging

Table 3 shows a comparison of the pathological outcomes of unilateral prostate lobes not suspected to harbor tumor preoperatively between the D’Amico low-risk group and D’Amico moderate to high-risk group. As can be expected, the pathologic stage and grade as well as tumor volume were observed to be more favorable among the low-risk group compared with the intermediate- to high-risk group. However, even among the 115 D’Amico low-risk group, the absence rate of malignancy was only 26.1%. Also the rate of Gleason 6 PCa was 30.4%.

**Table 3:** Comparison of pathological outcomes of unilateral lobe not suspected to harbor PCa by the combination of TRUS biopsy and mpMRI between D’Amico low risk group and D’Amico intermediate to high risk group.

Characteristics	D’Amico Low (n = 115)		D’Amico Intermediate to High (n = 220)		p-value
	Value	%	Value	%	
No malignancy	30	26.1%	16	7.3%	<0.01
Pathological Stage					
T2	83	72.2%	196	89.1%	
T3a	1	0.9%	5	2.3%	
T3b	1	0.9%	3	1.4%	<0.01
Pathological Gleason score					
3 + 3	35	30.4%	40	18.2%	
3 + 4	33	28.7%	97	44.1%	
4 + 3	16	13.9%	64	29.1%	
4 + 4	1	0.9%	3	1.4%	<0.01
Tumor volume (mL)					
Global tumor volume					
Median (range)	0.86 (0.03–9.8)		0.86 (0.03–9.8)		
<0.5, n (%)	48	41.7%	45	20.5%	
≥0.5, n (%)	37	32.2%	159	72.3%	<0.01
Main tumor volume					
Median (range)	0.61 (0.03–20.7)		0.61 (0.03–20.7)		
<0.5, n (%)	52	45.2%	80	36.4%	<0.01
≥0.5, n (%)	33	28.7%	124	56.4%	
Number of tumors					
1	43	37.4%	54	24.5%	
≥2	42	36.5%	150	68.2%	<0.01

Pca; prostate cancer, TRUS; transrectal ultrasonography, mpMRI; multiparameteric magnetic resonance imaging

Table 4 summarizes the pathological characteristics of unilateral prostate lobes not suspected to harbor tumor preoperatively by TRUS-guided biopsy and mpMRI. Among the 335 total subjects, unfavorable pathology and significant cancer were not observed in 36.1% and 30.7%, respectively. The absence rates of unfavorable pathology and significant cancer, among the D’Amico low-risk group were 56.5% and 47.8%, respectively. Meanwhile, the absence rates of dominant Gleason pattern 4 or 5 were 74.9% and 85.2%, respectively, among the 335 total subjects and the low-risk group only.

**Table 4:** Absence rates of significant cancer, unfavorable disease, and dominant Gleason pattern ≥ 4 according to the subgroups.

		Whole Group (n = 335)	D’Amico Low (n = 115)	D’Amico Intermediate to High (n = 220)	Low vs Intermediate to High (p-value)
Pathology Result of Non-suspicious Lobe From RP Specimen	Absence of Malignancy	46 (13.7%)	30 (26.1%)	16 (7.3%)	<0.01
	Absence of Significant Cancer	103 (30.7%)	55 (47.8%)	48 (21.8%)	<0.01
	Absence of Unfavorable Pathology	121 (36.1%)	65 (56.5%)	56 (25.5%)	<0.01
	Absence of Dominant Gleason pattern 4 or 5	251 (74.9%)	98 (85.2%)	153 (69.5%)	<0.01

RP; radical prostatectomy

Table 5 demonstrates the sensitivity analysis outcome of the approach that cominbed TRUS biopsy and mpMRI (positive biopsy core or PI-RAD≥3 lesion) in predicting the overall PCa, significant cancer, and unfavorable pathology in each prostate lobe.

**Table 5:** Sensitivity analysis outcome of the combination of TRUS biopsy and mpMRI (positive biopsy core or PI-RAD≥3 lesion) to detect malignancy, significant cancer, and unfavorable pathology of each prostate lobe (670 lobes) among study cohort (n = 335).

	Sensitivity %, (95% CI)	Specificity %, (95% CI)	Positive Predictive Value %, (95% CI)	Negative Predictive Value %, (95% CI)	Positive Likelihood Ratio (95% CI)	Negative Likelihood Ratio (95% CI)
Malignancy	52.9% (48.9% to 56.9%)	82.1% (69.6% to 91.1%)	95.8% (94.9% to 98.3%)	13.7% (12.1% to 15.6%)	2.96 (1.68 to 5.22)	0.57 (0.49 to 0.66)
Significant Cancer	55.5% (51.1% to 59.8%)	69.1% (61.1% to 76.4%)	86.3% (83.0% to 89.0%)	30.8% (27.8% to 33.9%)	1.80 (1.40 to 2.31)	0.64 (0.56 to 0.74)
Unfavorable Pathology	56.4% (51.9% to 60.9%)	67.6% (60.2% to 74.4%)	82.7% (79.2% to 85.7%)	36.1% (32.9% to 39.5%)	1.74 (1.39 to 2.18)	0.64 (0.56 to 0.74)

TRUS; transrectal ultrasonography, mpMRI; multiparameteric magnetic resonance imaging

## Discussion

In this study, we observed that only about 14% of the potential candidates for hemi-ablation actually had true unilateral disease on final pathologic analysis of RP specimens. Moreover, although not many, some candidates even harbored locally advanced disease. Even when the analysis was limited to subjects in the low-risk group (PSA<10 ng/ml, Gleason 6 or less and T1c/T2a), only 26.1% were found to have pathologically-confirmed unilateral disease among the potential candidates for hemi-ablation. Even if the goal of hemi-ablation was only to remove of significant cancer or unfavorable pathology, such a goal would have only been achieved in about one third of our potential candidates for hemi-ablation. Moreover, even when limited to only those in the D’Amico low risk group, about half of the potential candidates for hemi-ablation were found to harbor unfavorable pathology or significant cancer in the unilateral lobe not suspected to harbor tumor preoperatively. Overall, the current study demonstrated that the combination of extended TRUS-guided prostate biopsy and mpMRI did not provide reliable accuracy in the prediction of unilateral PCa, which is essential in the identification of appropriate candidates for hemi-ablative focal therapy.

Despite the appeal of performing organ-sparing treatment for PCa, concerns regarding the risk of failing to detect the significant tumor foci in untreated prostatic region largely limit the more widespread use of focal therapy. As widely known, PCa is often multifocal with incidence ranging from 67% to 87% [15]. On the other hand, it has been reported 20% of PCa patients have completely unilateral disease with small tumor volume [16]. Such cases can be considered appropriate for hemi-ablative focal therapy. Today, the widespread use of PSA screening has led to more men being diagnosed with PCa at earlier stage with smaller volume than ever [1]. As such, suitable candidates for focal therapy may increase as well. It would be important to develop tools for accurate detection of tumor foci in the prostate.

Several recent reports have offered promising findings on the accuracy of MRI in the depiction and localization of tumor foci in the prostate [17–20]. Others have reported that MRI may indeed be helpful in avoiding unnecessary biopsies [21]. A meta-analysis revealed that mpMRI has a sensitivity of 66–81% and specificity of 82–92% for PCa detection [7]. Such moderate sensitivity and high specificity of contemporary mpMRI for significant or index lesions and the ability to anatomically localize these lesions indicate that mpMRI may indeed be helpful tool in the selection of candidates for focal therapy. Nonetheless, studies evaluating the accuracy of mpMRI for the identification of unilateral PCa are lacking in the literature. Matsouka et al reported that diffusion-weighted MRI and a combined TRUS/transperineal biopsy technique with 14 cores showed a negative predictive value of 95.7% for predicting lobes with significant tumor foci [22]. It should be noted that insignificant PCa included Gleason score 3 + 4 PCa in their study. Tran et al evaluated the accuracy of mpMRI and transperineal template-guided mapping biopsy for identifying the locations of significant PCa in 50 subjects, they found that sensitivity, specificity, and negative predictive value for such combination for localizing the significant disease were 97%, 61%, and 91%, respectively [23]. A more recent multicenter prospective study—funded by UK government—also demonstrate the significant correlation between mpMRI and pathological outcome of transperineal template-guided biopsy, showing the ability to rule out significant cancer with high accurary, with 93% sensitivity and 89% negative predictive value [24]. The findings from these studies suggested that mpMRI could be useful in the selection of candidates for hemi-ablative focal therapy by providing a high negative predictive value in the detection of significant PCa.

On the other hand, not all published reports have offered such promising data on the accuracy of mpMRI. Though not on mpMRI, Jeong et al investigated the accuracy of prostate biopsy and conventional MRI in predicting the laterality of PCa, and found only a fair correlation between the combination approach (biopsy and conventional MRI) and laterality of PCa [25]. In their series of men who underwent preoperative MRI and subsequent RP, the positive predictive value for predicting pathologic unilaterality was 34.8%, using an approach that combined extended TRUS-guided biopsy and conventional MRI. Analyzing patient who underwent preoperative mpMRI before RP, Rosenkrantz et al reported that 36% of cancers that were underdetected with mpMRI had a Gleason score of ≥ 7 [26]. Moreover, a study on pathologic analyses of RP specimen in patients who had negative finding from preoperative mpMRI found that 61.4% of subjects had a pathologic Gleason score ≥ 7 [27]. These data on the rate of pathologically-confirmed significant cancers undetected by preoperative mpMRI would be supportive of the results from our study. Although recent reports have shown a relatively high accuracy of mpMRI in the localization of intra-prostatic tumor foci, there remains controversy, as can be seen from our findings as well as from other previous studies.

Several factors may have contributed to such a discrepancy. First, most of the published studies on mpMRI in men who underwent RP included only a small number of highly selected subjects [22, 23]. In such studies, mpMRI was mostly performed prior to biopsy, which was not the case in this present study. Currently, MRI is still more frequently performed for staging purposes following tissue confirmation of PCa from biopsy rather than in pre-biopsy setting. Pre-biopsy mpMRI may be associated with the possibility of selection bias, as it can influence the decision on whether to perform prostate biopsy or not. Although our study was retrospective in nature, it depicts a real-life situation on a relatively larger cohort of consecutive patients managed at our institution. Second, the difference in biopsy approach should be considered. Our patients underwent contemporary TRUS-guided biopsy, whereas other studies included patients who received transperineal mapping or template-guided biopsy. It has been reported that less than 40% of patients with unilateral disease on TRUS-guided biopsy actually had pathologically-confirmed unilateral disease on RP specimens [28]. Tumor foci not detected by TRUS-guided biopsy tend to be located more commonly in the anterior prostate than in the posterior prostate [29]. Recent studies showed that newer biopsy approaches, such as transperineal template-guided biopsy, enabled more efficient detection of tumor foci, resulting in superior accuracy [30, 31]. Since template-guided mapping biopsy offers systematic sampling of the entire prostate, it is considered to be the diagnostic tool for focal therapy. However, it cannot be denied that TRUS-guided biopsy, compared with newer biopsy techniques, is still more commonly performed in the actual clinical setting. Additionally, regardless of the version or type of MRI scanners used, the inter-observer variability should always be considered in studies on imaging findings [32]. Meanwhile, for our subjects, mpMRI was mostly performed with state-of-the-art 3-T scanners and analyzed by expert radiologists with more than 20 years of clinical experience. Overall, it can be stated that our study may be more reflective of true real-life contemporary clinical practice than many other relevant studies.

Our findings from real-life clinical practice revealed that the combination of contemporary extended TRUS-guided biopsy and mpMRI did not predict unilaterality of PCa with reliable accuracy. Therefore, the benefit of performing a repeat mpMRI-guided biopsy after the initial TRUS-guided biopsy, for the purpose of improving the accuracy of laterality prediction, such as in-bore biopsy, cognitive fusion or MRI/US fusion biopsy, might be limited. The mpMRI-guided biopsy before the initial biopsy could improve accuracy; however, to the best of our best knowledge, there is no published study on the specific subject. At this time point, a more accurate biopsy scheme is needed for the proper selection of candidates for hemi-ablative focal therapy. Although transperineal template-guided mapping biopsy and saturation biopsy approach may be more costly and likely to increase the level of patients’ burden and discomfort, such a scheme provides more precise representation of the intra-prostatic lesion. These new biopsy strategies should be thoroughly evaluated and refined to improve efficiency and safety for a wide application.

In the light of recent studies, mpMRI appears to be a promising modality for localization of tumor foci. However, real-time monitoring of focal therapy seems to be technically challenging. For real-time monitoring of focal therapy, newer multiparametric ultrasonography, such as shear wave elastography and contrast-enhanced ultrasound, may well be more appropriate in the future. [6]

The present study has some limitations to consider. The retrospective design may be a limiting factor to our data. Moreover, only patients who underwent definitive treatment of radical prostatectomy were included in the study, which may have introduced selection bias. However, for a series on preoperative mpMRI in a RP cohort, the number of subjects in our study is relatively larger than or comparable to other similar series. Meanwhile, clinicopathologic profiles of our subjects appeared to be relatively more aggressive (higher PSA level, higher rate of high-grade disease) than those reported from western series. The rate of PSA screening in Asia is still not as high as in Western countries [33]. Although there is an on-going trend of decreasing gap, PCas in Asian men are diagnosed at relatively higher PSA, higher Gleason score, and higher stage than in their Western counterparts.

## Conclusions

In this study, we observed that the combination of multi-core TRUS-guided prostate biopsy and mpMRI did not provide reliable accuracy in predicting the true unilaterality of PCa in patients who underwent prostate biopsy, preoperative mpMRI, and RP. This suggests that improvement on biopsy and MRI technique is warranted for an accurate localization of tumor foci, which is crucial for the appropriate selection of candidates for focal therapy.
